# Co-infection of canine parvovirus and circovirus in fatal gastroenteritis outbreak among service dogs in Kazakhstan, 2023

**DOI:** 10.3389/fcimb.2025.1645697

**Published:** 2025-09-22

**Authors:** Temirlan Sabyrzhan, Marat Kumar, Aidyn Kydyrmanov, Yermukhammet Kassymbekov, Nailya Klivleyeva, Baiken Baimakhanova, Kobey Karamendin

**Affiliations:** ^1^ The Laboratory for Ecology of Viruses, Research and Production Center for Microbiology and Virology, Department of Virology, Almaty, Kazakhstan; ^2^ Laboratory of Biochemistry of Viruses, Research and Production Center for Microbiology and Virology, Department of Virology, Almaty, Kazakhstan; ^3^ Department of Microbiology, Deputy Director of Research and Production Center for Microbiology and Virology, Almaty, Kazakhstan

**Keywords:** dog, kennel, canine parvovirus, canine circovirus, next generation sequencing, mortality

## Abstract

**Introduction:**

Between November 2023 and January 2024, a severe gastroenteritis outbreak with high mortality occurred among working dogs based in the Almaty region of Kazakhstan. The epidemic was characterized by an acute onset, rapid progression, and resulted in the death of over 100 juveniles (under 12-month-old) and several vaccinated adult dogs. In this study, we investigated the co-occurrence of canine circovirus and canine parvovirus DNAs in clinical samples from affected dogs, performed genetic characterization of the identified viruses, and evaluated their role in the outbreak.

**Methods:**

Polymerase Chain Reaction and Massive Parallel Sequencing methods were used in this research.

**Results:**

Polymerase chain reaction analysis of clinical samples revealed the presence of canine parvovirus in eight of the ten samples examined. Further, high-throughput sequencing of pooled oral, rectal, and blood swabs revealed that the majority of viral sequences corresponded to viruses in the Circovirus genus (*Circoviridae*, 42.3%), followed by Protoparvovirus genus (*Parvoviridae*, 38%), together accounting for over 80% of all viral reads.

**Discussion:**

Viral co-infections are a leading cause of mortality in dogs, with canine parvovirus enteritis often complicated by other pathogens such as canine distemper virus, canine coronavirus, and rotavirus. The presence of multiple pathogens can obscure the primary etiology, highlighting the need for comprehensive molecular diagnostics. Our findings underscore the critical importance of advanced molecular diagnostics in resolving complex infectious disease outbreaks in canine populations and inform future strategies for outbreak prevention and control.

## Introduction

Canine infectious diseases represent a persistent and urgent threat to both domestic and working dog populations worldwide, with outbreaks of highly contagious pathogens such as canine parvovirus (CPV), canine distemper virus (CDV), and canine circovirus (CanineCV) frequently resulting in significant morbidity and mortality, especially among young or immunologically naïve animals. Co-infections and viral recombination commonly occur in high-risk canine populations, such as those in shelters and among working dogs, posing additional challenges for accurate diagnosis and effective prevention (Decaro and Buonavoglia, 2012). The rapid transmission dynamics of these viruses, exacerbated by high-density environments such as kennels and shelters, can lead to explosive outbreaks with case fatality rates exceeding 90% in unvaccinated puppies (Godsall et al., 2010; Miranda and Thompson, 2016). Moreover, studies have shown that parvovirus infections can persist subclinically in partially immunized or maternally protected dogs, allowing silent viral shedding and subsequent spread (Elia et al., 2015). Additionally, the emergence of novel viral strains and co-infections further complicates diagnosis and control, as seen in recent reports of CPV and CanineCV co-circulation, which have been associated with increased disease severity and vaccine breakthrough cases (Faraji et al., 2022). The continued genetic diversification of these pathogens, coupled with factors such as maternal antibody interference and incomplete vaccination coverage, underscores the critical need for enhanced surveillance, rapid molecular diagnostics, and optimized vaccination strategies to mitigate the impact of canine infectious diseases on animal health and welfare (Decaro and Buonavoglia, 2012; Day et al., 2016).

CPV belongs to the genus *Protoparvovirus* in the *Parvoviridae* family and is considered to be one of the smallest viruses known (Chung et al., 2020; Singh et al., 2021). Members of the *Parvoviridae* family can infect various species of mammals, including domestic dogs, raccoons, cats, coyotes, wolves, and marine mammals such as seals. The virus is widespread in the environment and can remain viable for more than a year under suitable conditions (Mylonakis et al., 2016; Karamendin et al., 2024). Dogs with CPV are more susceptible to co-infection with various pathogens (Anderson et al., 2017). In turn, circovirus replication is enhanced in tissues undergoing cellular regeneration, such as those damaged by CPV-induced necrosis (Thaiwong et al., 2016). Circoviruses are usually detected in association with parvoviruses, which are often associated with the occurrence of enteritis. Dual infections, mainly attributed to CPV-2, suggest their synergy in disease development (Hsu et al., 2016).

CanineCV is a non-enveloped, icosahedral, single-stranded, covalently closed circular DNA virus of ≈2 kb in size. It is a species in the family *Circoviridae* (Li et al., 2013; Giraldo-Ramirez et al., 2020). CanineCV was first identified in the United States in 2012 (Kapoor et al., 2012). In dogs, infection is associated with vasculitis, hemorrhaging, hemorrhagic enteritis, and diarrhea (Niu et al., 2020). Recent studies have suggested that CanineCV may contribute to the severity of disease in coinfections by modulating host immunity. The *Rep* Canine CV protein suppresses the host immune response, thereby facilitating the replication of CPV-2. This may exacerbate the clinical manifestations of coinfection in dogs (Hao et al., 2022).

This study documents a fatal outbreak of canine gastroenteritis in Kazakhstan, characterized by co-infection with CPV and CanineCV, and highlights the importance of advanced molecular diagnostics in outbreak investigations. To date, no studies have reported on the molecular characteristics of CPV and CanineCV co-infections in Central Asia. Therefore, this study also provides new regional insights into the evolution and circulation of these viruses in previously uncharacterized dog populations.

## Materials and methods

Laboratory and field studies were carried out in compliance with all bioethical standards in accordance with the instructions of the local ethics commission №02-09–130 from 20.10.2022.

### Collection of animal samples

Clinical samples, including oropharyngeal and rectal swabs and blood serum, were collected from a total of 10 dogs (both symptomatic and asymptomatic). Due to late reporting, clinical material was only obtained from symptomatic dogs housed in the facility’s quarantine unit. The facility’s remaining animals were either asymptomatic or had already died, precluding further sampling. All these factors, unfortunately, did not allow us to collect more samples from dogs. Selection included three juveniles under 6 months of age, three neutered males, and four unsterilized females. Each animal was double-swabbed to enable molecular diagnostic screening. For molecular diagnostics, swabs were placed in DNA/RNA Shield reagent (Zymo Research, USA) and stored at ambient temperature until processing. Duplicate swabs were preserved in viral transport media and archived at -80 °C for future analyses.

### Dog breed representation

**Table 1** provides breed diversity of sampled dogs: four Belgian Shepherds and four German Shepherds with different vaccination histories and vaccinated single representatives of *Dutch Shepherd* and *Kazakh Tazy*. PCR-positive samples were pooled for further library preparation.

**Table 1:** Sampled dog breed representation.

№	Breed	Age	Sex	Vaccinated by	Symptoms	PCR positive
1	Dutch Shepherd	3 months	Male	Nobivac DP	Diarrhea, vomiting, anorexia, dehydration	CPV
2	Belgian Shepherd	2 months	Male	Nobivac DP	Hematochezia, vomiting, anorexia, dehydration	CPV, Rotavirus
3	Belgian Shepherd	1 month	Male	–	Hematochezia, vomiting, lethargy	CPV
4	Belgian Shepherd	2 years	Female	Nobivac DP	Lethargy	CPV
5	German Shepherd	6 years	Male	Nobivac DP	Lethargy	CPV
6	German Shepherd	10 months	Male	Nobivac DP	Diarrhea, vomiting, anorexia, dehydration	CPV, Rotavirus
7	German Shepherd	3 years	Male	Nobivac DP	Lethargy	CPV
8	Kazakh Sighthound (Tazy)	6 months	Female	Nobivac DP	–	CPV
9	German Shepherd	5 years	Female	Nobivac DP	–	–
10	Belgian Shepherd	6 months	Female	Nobivac DP	–	–

### Vaccination history

Until 2023, the kennel used the combined live attenuated vaccine Nobivac^®^ DP (MSD Animal Health) for prophylaxis against CDV and CPV. Subsequently, the vaccination protocol was updated to the Biocan Novel DHPPi (Bioveta, CZ), a polyvalent live vaccine against parvovirus, CDV, infectious hepatitis, infectious laryngotracheitis, and parainfluenza.

While adult dogs were reportedly vaccinated in accordance with the manufacturer’s standard protocols, juveniles were vaccinated earlier than recommended due to the urgent spread of the disease. This crisis-driven decision was made by facility personnel in an attempt to control viral transmission.

### Reverse transcription and polymerase chain reaction

PCR/RT-PCR screening was performed using the OneTaq One-Step PCR kit (New England Biolabs, USA) with diagnostic primers targeting CDV, Influenza A virus (IAV), canine coronaviruses (CCoV), rotavirus, and canine parvovirus. Primer sequences and cycling conditions followed published protocols (Senda et al., 1995; Jensen et al., 2002; Payungporn et al., 2004; Moës et al., 2005; Ortega et al., 2017). Briefly: DNA/RNA extraction was performed using the QIAamp Viral RNA Mini Kit (Qiagen, Germany) (QIAGEN, 2015) following the manufacturer’s instructions. Amplification of the targeted genes of viruses with a concentration of 0.5 mM each primer in a 25 μL final volume of PCR mix. The reactions were performed in an Eppendorf Gradient thermocycler.

### Genome library preparation and sequencing

Libraries were prepared using the QIAseq FX Single Cell RNA Library Kit (Qiagen, Germany) and NEBNext Ultra DNA Library Prep Kit for Illumina (NEB, USA). cDNA fragmentation (450–500 bp) was performed using the NEB Fragmentase kit (NEB, USA). Adenine overhangs and Illumina-compatible adapters were ligated, and libraries were purified and amplified per the manufacturer’s protocols. Illumina v.3 chemistry kit (2 x 300 cycles) was used for sequencing on the MiSeq sequencer (Illumina, USA).

### Bioinformatic analyses

Raw reads were processed using the LAZYPIPE pipeline (Plyusnin et al., 2020) that included initial quality control and adapter/low-quality base trimming with Trimmomatic v0.39. Host-derived reads were filtered using BWA-MEM v0.7.17. The remaining reads were *de novo* assembled with MEGAHIT v1.2.9 (–presets meta-sensitive), and analyzed on a high-performance computer with Geneious Prime software (Biomatters, New Zealand). Homology searches were performed using BLASTn and BLASTx against GenBank’s non-redundant and viral reference databases (E-value <10^-25^).

Alignment and phylogenetic analyses were performed in MEGA X (neighbor-joining, 500 bootstrap replicates, Tamura-Nei model) (Kumar et al., 2018) based on complete genome sequences. Donut charts visualizing viral family distributions were generated in R (v4.2.0) with ggplot2 (Wickham, 2016). Comparative ORF analyses were performed in Geneious (v2025.1). All trees were constructed based on the complete genomic sequences presented in the NCBI database to assess the phylogenetic relationships of the canine circovirus KZ_2024 and canine parvovirus KZ_2024 strains obtained in this study.

## Results

### Outbreak description, clinical presentation, and PCR screening

In September 2023, an outbreak of acute gastroenteritis occurred among high-breed service dogs in suburban Almaty, Kazakhstan. Clinical signs included lethargy, seizures, anorexia, vomiting, and hemorrhagic diarrhea, with a high case fatality rate. By the end of November 2023, a total of 110 dogs were affected, resulting in significant mortality, particularly among juveniles.

**Table 2** summarizes the PCR screening results. All samples tested negative for IAV, CDV, and CCoV. Parvovirus was detected in 8 swabs, while two dogs tested positive for rotavirus. Seven of eight PCR-positive for CPV dogs were vaccinated (**Table 1**).

**Table 2:** PCR screening results.

Pathogen	Positive samples	Total samples	Percentage (%)
Parvovirus	8	10	80
Rotavirus	2	10	20
IAV	0	10	0
CDV	0	10	0
CCoV	0	10	0

### Next-generation sequencing and virome composition

Illumina MiSeq sequencing yielded 3,274,832 raw reads, with 2,968,286 retained after quality filtering. *De novo* assembly produced 20,158 contigs (mean length: 502 nt; median: 360 nt), which were aligned to local viral protein databases.

**Figure 1:**
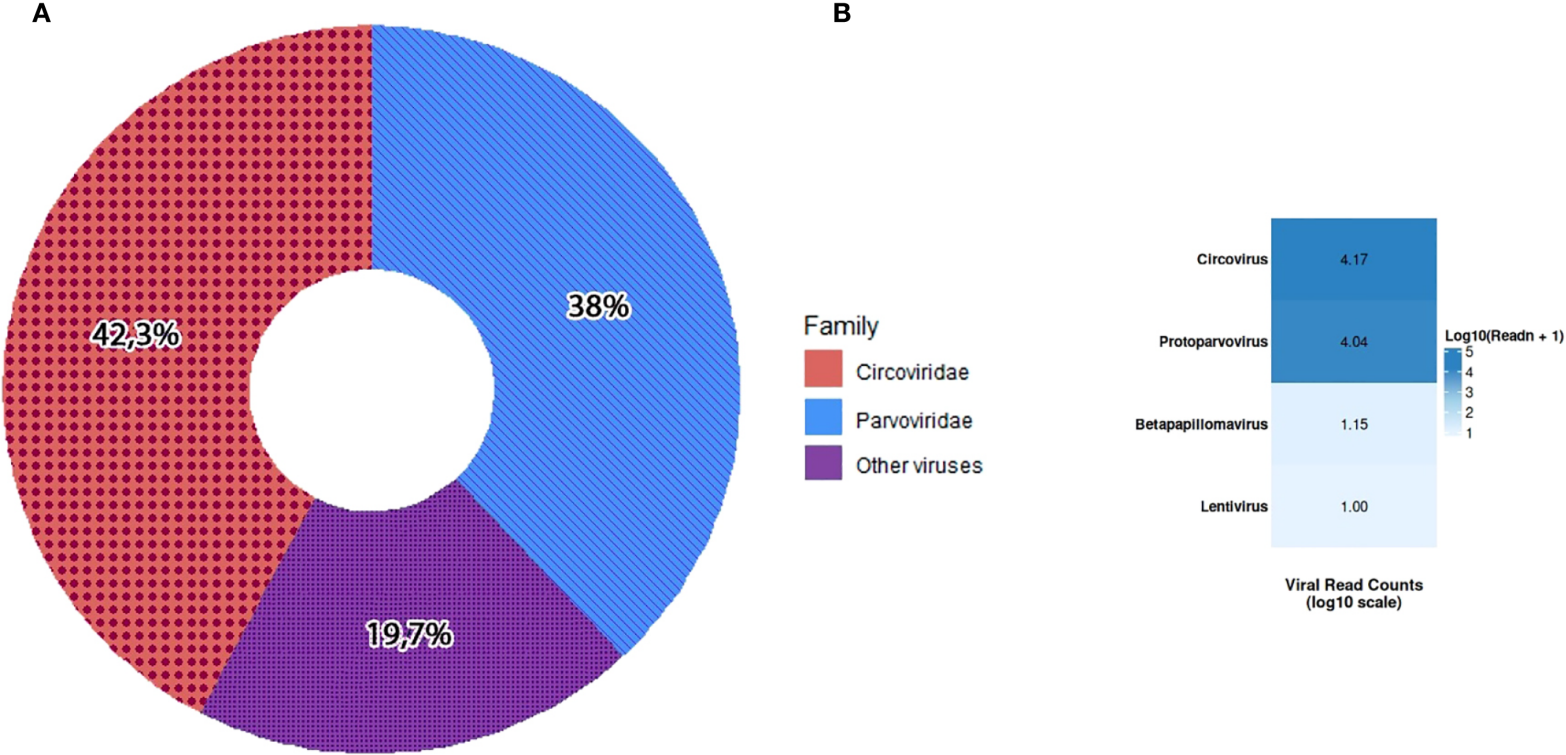
Distribution of viral family sequence reads **(A)**. Heatmap of relative read counts for each viral genus detected by NGS **(B)**. Color intensity reflects normalized read abundance (darker = more reads).

The final consensus sequence for KZ_2024 circovirus was 2,063 nt in length, supported by 14,453 mapped reads (42.3%), while KZ_2024 parvovirus consensus was 5,058 nt in length, supported by 12,961 mapped reads (38%), together accounting for over 80% of all viral reads (**Figure 1A**). This distribution is consistent with previous studies of the sick canine enteric virome (Moreno et al., 2017; Esposito et al., 2022).

The visualization (**Figure 1B**) highlights that the *Circoviridae* and *Parvoviridae* families have the strongest signals (dark blue) in most samples, consistent with the co-infection pattern. Other viruses, such as betapapillomavirus and lentivirus, were less represented in the analysis.

### Phylogenetic analyses

**Figure 2:**
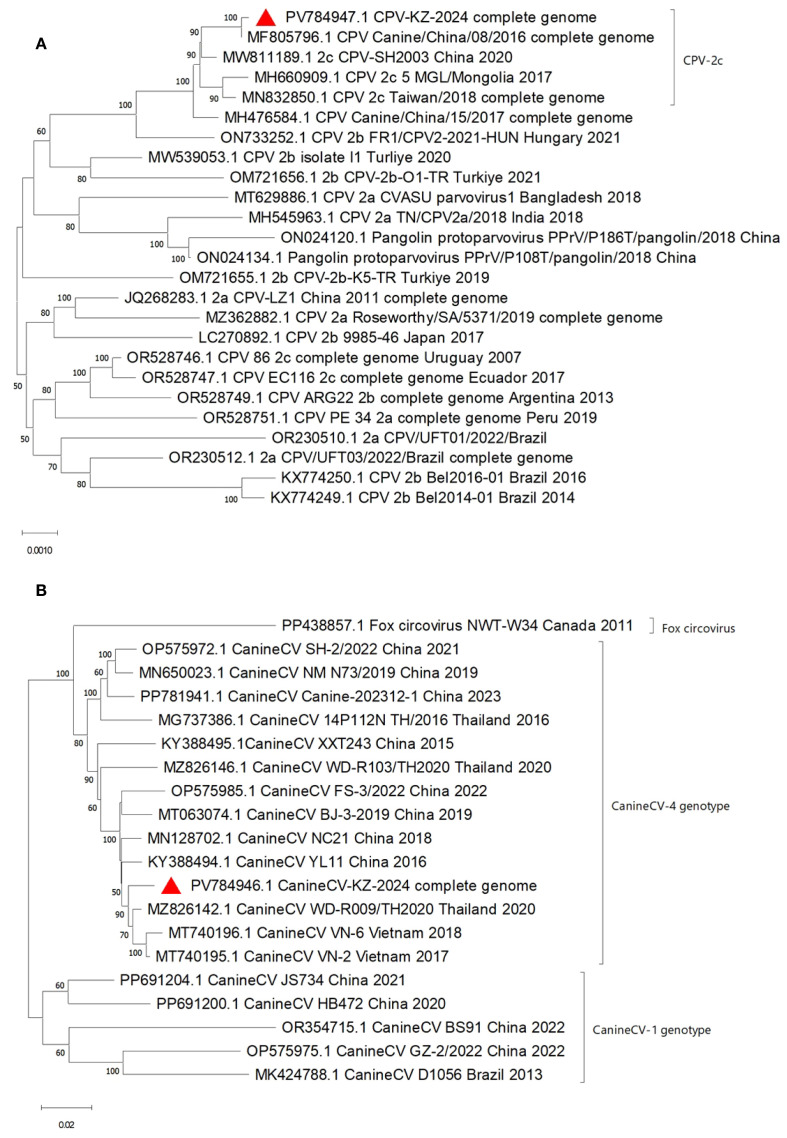
Phylogenetic Tree of Canine Parvovirus KZ_2024, 5,058 nt complete genome **(A)** and Canine Circovirus KZ_2024, 2,063 nt complete genome **(B)**.

Despite the geographical distance, the KZ_2024 parvovirus strain’s complete genome exhibited high genetic similarity to the 2016CPV strains’ genome from China (MF805796), suggesting either a stable phylogenetic lineage or a recent introduction from neighboring countries. The data support CPV-2c as the dominant variant in the region. The KZ_2024 circovirus strain formed a distinct clade with Southeast Asian strains (Thailand: MZ826142; Vietnam: MT740195, MT740196) and was clearly separated from most circoviruses (e.g., MT063074) from China (**Figures 2A, B**).

### Genome organization of detected viruses

#### Genomic analysis of canine circovirus KZ

Sequence analysis revealed a high degree of similarity (>99% nucleotide identity) between the Canine circovirus KZ strain and previously described CCV genomes. The complete genome consists of 2,063 nucleotides with a GC content of 50.6%. It contains two predicted open reading frames (ORFs) transcribed from opposite DNA strands, as well as two intergenic regions. These ORFs encode the viral replicase (Rep) and capsid protein (Cap), comprising 269 and 303 amino acids, respectively. Four unique amino acid substitutions were identified in the *Cap* gene and one in the *Rep gene* that were not observed in other available CCV sequences, suggesting potential strain-specific adaptations.

The *Cap* gene shows a balanced nucleotide composition, with a GC content of 53.2% and AT content of 46.8%. Codon position analysis indicates a preference for guanine in the first position (33.6%) and adenine in the second (32.6%). The *Rep gene* spans 809 nucleotides and displays a moderate AT bias (52.5%), with adenine (31.3%) being the most prevalent nucleotide. This codon usage pattern is consistent with that observed in other global CCV isolates and may reflect evolutionary trends or mutational pressures.

#### Genomic analysis of canine parvovirus KZ

The Canine parvovirus KZ strain also demonstrates high sequence similarity to previously reported CPV genomes. Its complete genome comprises 5,058 nucleotides with a GC content of 35.7%, and contains multiple ORFs encoding structural and non-structural proteins, including *NS1* and *VP2*. The *NS1* gene encodes a 668-amino acid non-structural protein, while the *VP2* gene encodes the major capsid protein with 584 amino acids.

The *VP2* gene is 1,755 nucleotides long and is characterized by a high AT content (64.3%), with adenine (35.2%) and thymine (29.1%) dominating, particularly in the third codon position (A – 40.5%, T – 44.3%). This pattern aligns with the codon usage observed in Asian CPV-2c strains. The *NS1* gene spans 2,007 nucleotides and also exhibits a strong AT bias (64.2%), with adenine being the dominant nucleotide (38.0%). Compared to reference sequences, several unique amino acid substitutions were identified in the *VP2* gene of the KZ strain, potentially contributing to its genetic differentiation from previously reported isolates.

### Comparative analysis of mutations

Genetic analysis of the VP2 gene of the Kazakhstan strain of canine parvovirus (KZ) revealed the following substitutions: 370 (Q → R), 426E (N → E) — the key substitution — and 440 (A → T), which are all characteristic of the CPV-2c variant (Fu et al., 2022) (**Supplementary Table S1**). In the NS gene at position 60, a substitution I → V is observed, a characteristic of the CPV-2c strain (**Supplementary Table S2**). Similar substitutions have been observed in the reference strains from China and Vietnam. The substitutions that remain in the other positions of the isolated strain (Y544F, E545V, L630P) also correspond to the CPV-2c variant (Wang et al., 2016). The CanineCV genome encodes two major proteins: the conserved Rep, which is responsible for replication, and Cap, a variable structural protein (Kotsias et al., 2019). Analysis of the Cap gene (**Supplementary Table S3**) revealed a combination of mutations that partially coincided with isolates from China and Thailand at positions 13, 16, 29, 58, 79, 95, and 101. This finding indicates significant differences from European and American strains and demonstrates phylogenetic proximity to Asian strains. Concurrently, no alterations were detected in the pivotal immunogenic positions 24R, 50V, 103R, and 111R, suggesting antigenic conservatism. Position 149G, which is considered rare, has been hypothesized to be a marker of a unique subvariant or point adaptation. A unique mutation, designated Q8H, was identified in the Rep gene (**Supplementary Table S4**), along with 71C, a characteristic that was exclusively observed in the Kazakh and Thai lines. Despite the phylogenetic proximity to the Asian lines, we have revealed mutations 115R and 149Y, which are characteristic of the European and American lines.

## Discussion

Gastroenteritis is common in domestic carnivores, particularly in animals under one year of age, especially in areas with high animal densities, such as kennels and animal shelters (Capozza et al., 2023). In our study, the affected animals ranged in age from 1 month to 6 years, with the majority being juvenile dogs under 12 months old. Canine diseases with similar symptoms can be caused by CanineCV (Decaro and Buonavoglia, 2011), CPV, and rotavirus (Malik et al., 2020). CPV and canine enteric coronavirus (CECoV) are considered the most common viral agents causing gastroenteritis in dogs (Decaro et al., 2011).

In various studies of the virome of domestic animals with signs of enteritis, in addition to CPV and CanineCV, astroviruses, rotaviruses, bocaviruses, and kobuviruses usually predominate. In the intestinal virome of healthy dogs, the majority of reads are usually bacteriophages, the majority (up to 80% or more) of which are *Myoviridae*, *Siphoviridae*, *Podoviridae*, and *Microviridae*. In dogs with severe diarrhea, other viruses (herpesviruses, retroviruses, etc.) can be detected at low levels as concomitant or background infections (Moreno et al., 2017; Esposito et al., 2022). In this research, the most abundant viral genera were *Circovirus* (42.3%) and *Parvovirus* (37.8%). Other detected viruses included betapapillomavirus and lentivirus, but they were less represented in the virome analysis. CPV was first detected in vaccinated dogs by PCR, and further high-throughput sequencing allowed us to distinguish the positives from vaccine strains by finding mutations not specific to vaccine strains in the virome. PCR-positive samples were pooled for further library preparation. In this regard, we were unable to compare the results obtained in PCR with data obtained by Next-Generation Sequencing.

The BLAST results showed that the CPV sequence we obtained is highly similar to three reference sequences registered in the NCBI database: MF134808.1, isolated from a dog in China in 2017 and belonging to the CPV-2 genotype; OR296281, a partial genome of the CPV-2a isolate CO2_2011, obtained from a fecal swab of a dog in Coimbatore (India) in 2009/2019; and MT165692, a CPV-2b strain, also isolated in China in December 2017. High identity (99.64%) and complete coverage (100%) indicate that our strain is genetically very close to widespread CPV strains, allowing us to assign it to the same phylogenetic lineages represented by both CPV-2a and CPV-2b.

Mutant strains of CPV-2a, CPV-2b, and CPV-2c are gradually pushing the original CPV-2 into the background (Ogbu et al., 2020). For example, the CPV-2a line is the dominant strain of parvovirus circulating in China (61.81% of isolates) (Zhuang et al., 2019). Mutation analysis has shown that our sequence belongs to the CPV-2c strain. Given the geographical proximity, the possibility of parvovirus B19 migration from China to Kazakhstan cannot be excluded. Additionally, service dogs participating in exhibitions and international competitions are at a higher risk of infection.

Comparative analysis with GenBank data showed that the highest similarity to our strain was shared by two canine circovirus strains from China (OQ198057 and OQ198058), obtained from fecal samples of dogs in 2020-2021, and two strains from Thailand (MZ826142 and MZ826143), isolated from nasopharyngeal samples of dogs with respiratory disease in 2020. The high degree of similarity to these isolates may indicate common evolutionary roots or similar routes of virus spread in these regions. Genetic analysis of the CanineCV revealed phylogenetic proximity to Asian lineages and distance from European ones.

Complete genome analysis revealed several amino acid substitutions that distinguish the outbreak strains. In the CPV VP2 gene, multiple unique mutations were identified that are absent from available GenBank records. Since VP2 plays a key role in host cell binding and antigenicity, these substitutions may affect viral properties and require further investigation (Decaro and Buonavoglia, 2012). The VP2 structural protein is responsible for tropism and adaptation to new hosts. Substitutions in its sequence affect the virus’s biological properties and also serve as a marker for identifying CPV type 2 variants (Li et al., 2017). The role of NS1 gene mutations remains unclear. Shaohan’s study suggests that substitutions at positions 60, 443, 544, 545, and 630 can affect virion replication and packaging (Li et al., 2022).

Similarly, the Canine circovirus KZ strain exhibited four unique amino acid substitutions in the *Cap* gene and one in the *Rep* gene, not observed in previously described CanineCV sequences. As *Cap* and *Rep* are essential for virus structure and replication, these findings indicate the genetic divergence of the KZ strain from other global isolates (Hsu et al., 2016). The obtained amino acid substitutions suggest potential strain-specific adaptations, but to date, the biological implications of these mutations have not been explored, and further experimental analyses are necessary. The role of mutations in the genes of these proteins has not been thoroughly studied (Liu et al., 2024). However, evolutionary changes in the Cap gene are partly determined by the immunity of the host animal (Dankaona et al., 2024). Being single-stranded DNA viruses, both CPV and CanineCV undergo mutations due to their lack of proofreading mechanisms (da Silva et al., 2025). This results in ongoing genetic diversification.

The detection of both CPV and CanineCV, along with rotavirus in a minority of cases, suggests that viral co-infection may have contributed to the severity and high mortality observed during the outbreak. Compared to previously described co-infection cases of CPV and CanineCV (Thaiwong et al., 2016), the strain in this study led to much more mortality, but clinical symptoms were similar. It was previously hypothesized that clinical symptoms in co-infected dogs are more severe than in those with single infections (Thaiwong et al., 2016). Supposedly, CanineCV was not merely an incidental finding and likely played a pathogenic role, particularly in synergy with CPV. This conclusion is supported by previous compelling molecular findings and established biological mechanisms (Thaiwong et al., 2016). The similarity of KZ_2024 CPV to CPV-2c and CanineCV strains prevalent in China and other parts of Asia indicates possible regional endemicity. However, these strains are now spreading globally, and their distribution patterns may vary depending on geographic location, sampling period, and commercial movements of dogs imported from abroad (Decaro et al., 2007; Decaro and Buonavoglia, 2012).

In dogs, rotaviruses, which are wheel-shaped, double-stranded RNA viruses of the *Reoviridae* family, chiefly affect juveniles under 12 weeks of age, producing watery diarrhea and dehydration associated with group A (G3P3) rotavirus infections. This condition is generally mild in adults but can be zoonotic, making fluid therapy and strict hygiene the mainstays of care (Ortega et al., 2017). Although metagenomic sequencing confirmation for the rotavirus-positive PCR result was not obtained, the finding was independently verified by the Central Reference Laboratory. An internal, unpublished report further supported this result.

Canine papillomaviruses encompass more than 25 DNA virus types spread across the *Lambda*-, *Tau*-, and *Chipapillomavirus* genera; transmission by direct contact leads to oral or cutaneous warts that usually self-resolve within weeks, although persistent lesions in immunosuppressed dogs may evolve into squamous cell carcinoma, and viral DNA can be found on apparently normal skin (Medeiros-Fonseca et al., 2023). Lentiviruses, by contrast, are not a major natural canine pathogen—only an isolated canine immunodeficiency virus from a leukemic dog has been described (Perk et al., 1992).

The presence of these additional viruses, even if not the primary cause of mass mortality, underscores the importance of comprehensive diagnostics in understanding the full spectrum of pathogens contributing to disease in complex outbreaks. Some of the co-infecting agents can exacerbate clinical signs, particularly in a high-density environment where animals may experience stress or have underlying parasite loads, further compromising their immune systems. This reinforces the critical importance of advanced molecular diagnostics in resolving complex infectious disease outbreaks in canine populations.

Other, non-viral factors also influence susceptibility to parvovirus enteritis: for example, stress from overcrowding and parasite burden may have also exacerbated the outbreak (Grellet et al., 2013); climatic factors also influence susceptibility. The risk of parvovirus infection in dogs increases significantly in spring, late autumn, and early winter. Seasonal changes in temperature and climate may explain the increased likelihood of both CPV (Qi et al., 2020) and other viral infections. Although it was not possible to perform *in situ* hybridization (ISH) and other histological studies in our case, it is extremely unlikely that the primary agent and cause of mass mortality was precisely the circovirus. Such cases are extremely rare (Liu et al., 2024), since canine circoviruses mainly act as secondary agents associated with CPV (Zaccaria et al., 2016).

The dog center where the outbreak occurred houses several hundred dogs, a factor that is thought to have contributed to the rapid spread of the infection. All dogs in the kennel were vaccinated with the combined polyvalent vaccine Nobivac DP Plus, which is designed to protect against CPV and is expected to provide reliable immunity when administered correctly. Despite claims that vaccines may not provide complete protection against the different CPV variants, numerous studies (Larson and Schultz, 2008; Glover et al., 2012; Reitzenstein et al., 2012) have shown that CPV-2-based vaccines provide reasonable protection against CPV-2a, CPV-2b, and CPV-2c when the appropriate vaccination schedules are followed (Decaro et al., 2020).

CPV is common in many places and can be transmitted from healthy carriers, contributing to the spread of infection in the pet population. Vaccination against parvovirus can induce a good immune response in most dogs; all service dogs were vaccinated in the first two months after birth, but their samples were PCR-positive for parvovirus. This is probably because vaccinations are too early, which may affect maternal antibodies, increasing the susceptibility of juveniles to virus infection (Mazzaferro, 2020).

Maternally derived antibodies (MDA) can block active immunization after the administration of CPV vaccines. Vaccination of puppies with MDA titers that prevent vaccination may result in failure to seroconvert due to neutralization of the vaccine virus antigen. There is a period known as the “window of susceptibility” or “immunity gap”, averaging 2–3 weeks, during which the MDA titer falls below that required for protection but is still capable of neutralizing the vaccine virus. During this period, puppies can be infected and sometimes develop diseases (Decaro et al., 2020). The “immune window” following vaccination is a temporal vulnerability when the body has not yet mounted a consolidated, protective response. This period necessitates meticulously designed, multi-dose primary series and booster campaigns to achieve and maintain durable immunity. Additionally, the continuous evolution of pathogens through mechanisms like antigenic drift and shift necessitates constant vaccine reformulation and strategic booster campaigns to counter immune evasion (Zimmermann and Curtis, 2019). The confluence of these challenges—a population with waning immunity facing a novel, highly transmissible variant—creates a dynamic landscape where vaccine efficacy is a fluid metric, not a fixed value.

Thus, our data indicate that the key role in the outbreak was played by the conditions of confinement, early vaccination, and additional risk factors (high density of animals in the nursery, possible genetic resistance to vaccination, and possible parasite invasion). The sequencing results obtained confirm the importance of comprehensive diagnostics and the need to monitor both the main infection, canine parvovirus, and the concomitant infection, canine circovirus. Based on the trends identified, a possible preventive measure could be a revision of the vaccination schedule for juveniles, taking into account the level of maternal antibodies, as well as an improvement in the conditions of confinement. These measures, together with regular screening and systematic surveillance, can reduce the risk of such outbreaks. Although genetic resistance to CPV vaccination occurs in approximately one dog in a thousand (Day et al., 2007), epidemiological data are lacking, and further prospective studies are needed to determine the prevalence of primary vaccine failure.

This study has some limitations.

The main limitation of this study is the relatively small sample size (n=10 dogs) compared to the total kennel population (more than 200 dogs). At the time of our investigation, only 10 symptomatic animals were available for sample collection. Most of the other affected dogs had either died or were unavailable.

An additional factor was time and resource constraints: the studies were conducted urgently during an outbreak, reducing the opportunities for more detailed or long-term monitoring of the disease situation. Taken together, these factors may limit the extrapolation of the results to the whole kennel population.

The absence of histopathological or ISH evidence to definitively establish the primary pathogenic role of CanineCV is also acknowledged as a limitation.

Although the hemagglutination inhibition (HAI) assay using porcine erythrocytes was performed, we considered the results to be due to the inconsistency in duplicates and did not include them in the article. Also, limitations may include the absence of virus isolation, vaccine efficacy testing, or measurement of maternally derived antibody titers.

## Data Availability

The data presented in the study are deposited in the Genbank repository, accession numbers: https://www.ncbi.nlm.nih.gov/nucleotide/PV784946 and https://www.ncbi.nlm.nih.gov/nucleotide/PV784947.
