# Supplementary material for: Co-infection of canine parvovirus and circovirus in fatal gastroenteritis outbreak among service dogs in Kazakhstan, 2023
Source: Front Cell Infect Microbiol. 2025 Sep 22;15:1645697. doi: 10.3389/fcimb.2025.1645697 (PMC12497800; doi:10.3389/fcimb.2025.1645697)
Supplement: Supplementary file 1 [file Table1.docx]

Supplementary Table S1. VP2 gene amino acid mutations of Canine Parvovirus KZ_2024 strain

| Strain | Origin | GenBank accession no. | Amino acid position | | | | | | | | |
| --- | --- | --- | --- | --- | --- | --- | --- | --- | --- | --- | --- |
|  |  |  | 5 | 267 | 297 | 300 | 305 | 324 | 370 | 426 | 440 |
| CPV-2 | China, 2016 | MF805796 | A | Y | A | G | Y | I | Q | N | A |
| CPV-2a | China, 2017 | MF134808 | G | Y | A | G | Y | I | R | E | T |
| CPV-2a | South Korea, 2017 | MK144545 | A | Y | A | G | Y | I | Q | N | A |
| CPV-2b | China, 2011 | JQ268284 | A | Y | A | G | Y | I | Q | D | A |
| CPV-2c | China, 2017 | MH476592 | G | Y | A | G | Y | I | R | E | T |
| CPV-2c | South Korea, 2017 | MK144544 | G | Y | A | G | Y | I | R | E | T |
| CPV-2c | Hungary, 2021 | OQ108896 | A | **F** | A | G | Y | **Y** | Q | E | T |
| Canine parvovirus KZ | | | **G** | **Y** | **A** | **G** | **Y** | **I** | **R** | **E** | **T** |
