# Supplementary material for: Co-infection of canine parvovirus and circovirus in fatal gastroenteritis outbreak among service dogs in Kazakhstan, 2023
Source: Front Cell Infect Microbiol. 2025 Sep 22;15:1645697. doi: 10.3389/fcimb.2025.1645697 (PMC12497800; doi:10.3389/fcimb.2025.1645697)
Supplement: Supplementary file 2 [file Table2.docx]

Supplementary Table S2. NS1 gene amino acid mutations of Canine Parvovirus KZ_2024 strain

| Strain | GenBank accession no. | Amino acid position | | | | |
| --- | --- | --- | --- | --- | --- | --- |
|  |  | 60 | 433 | 544 | 545 | 630 |
| CPV-2_China / 2016 | MF805796 | V | N | F | V | P |
| CPV-2a_China / 2017 | MF134808 | I | N | Y | E | L |
| CPV-2a_South Korea / 2017 | MK144545 | I | N | Y | E | L |
| CPV-2b_China / 2011 | JQ268284 | I | N | Y | V | L |
| CPV-2c_China / 2017 | MH476592 | V | N | F | V | P |
| CPV-2c_South Korea / 2017 | MK144544 | I | N | Y | E | L |
| CPV-2c­_Viet Nam / 2017 | MT106234 | V | N | F | V | P |
| Canine parvovirus KZ | | **V** | **N** | **F** | **V** | **P** |
