# Supplementary material for: Co-infection of canine parvovirus and circovirus in fatal gastroenteritis outbreak among service dogs in Kazakhstan, 2023
Source: Front Cell Infect Microbiol. 2025 Sep 22;15:1645697. doi: 10.3389/fcimb.2025.1645697 (PMC12497800; doi:10.3389/fcimb.2025.1645697)
Supplement: Supplementary file 3 [file Table3.docx]

Supplementary Table S3. CAP gene amino acid mutations of Canine Circovirus KZ_2024 strain

| CCV Strain | GenBank accession no. | Amino acid position | | | | | | | | | | | | | | | | | | | |
| --- | --- | --- | --- | --- | --- | --- | --- | --- | --- | --- | --- | --- | --- | --- | --- | --- | --- | --- | --- | --- | --- |
|  |  | 13 | 16 | 24 | 29 | 35 | 42 | 50 | 51 | 58 | 79 | 83 | 95 | 101 | 102 | 103 | 111 | 136 | 144 | 148 | 149 |
| CCV/Thailand/2020 | MZ826142 | **R** | A | R | **N** | L | T | V | K | Q | A | T | Y | F | I | R | **K** | T | T | I | **G** |
| CCV/Argentina/2016 | MK033608 | S | T | R | R | L | T | V | K | T | **T** | T | **F** | Y | I | R | R | **Q** | **C** | R | L |
| CCV/USA/2011 | KC241984 | S | A | R | R | L | T | V | K | T | A | I | Y | Y | V | R | R | T | T | R | L |
| CCV/Germany/2014 | KT283604 | S | T | R | R | L | T | V | K | A | A | I | Y | Y | V | R | R | T | S | I | L |
| CCV/China/2016 | MF797786 | S | T | R | **N** | L | T | V | K | Q | A | T | Y | F | I | R | R | T | T | I | **S** |
| CCV/USA/2015 | MF457592 | S | T | R | R | L | T | V | K | T | A | T | Y | Y | I | R | R | T | T | I | L |
| CCV/Italy/2013 | KT734823 | S | T | R | R | L | T | V | K | T | A | T | Y | Y | V | R | R | T | T | R | L |
| CCV/KZ_2024 | | **R** | **A** | **R** | **N** | **L** | **T** | **V** | **K** | **Q** | **A** | **T** | **Y** | **F** | **V** | **R** | **R** | **T** | **T** | **I** | **G** |
