# Supplementary material for: Co-infection of canine parvovirus and circovirus in fatal gastroenteritis outbreak among service dogs in Kazakhstan, 2023
Source: Front Cell Infect Microbiol. 2025 Sep 22;15:1645697. doi: 10.3389/fcimb.2025.1645697 (PMC12497800; doi:10.3389/fcimb.2025.1645697)
Supplement: Supplementary file 4 [file Table4.docx]

Supplementary Table S4. REP gene amino acid mutations of Canine Circovirus KZ_2024 strain

| CCV Strain | GenBank accession no. | Amino acid position | | | | | | | | | | | | | | | | | | | | | | | | | | | | | | | | | | | | | | | | | | | |  |
| --- | --- | --- | --- | --- | --- | --- | --- | --- | --- | --- | --- | --- | --- | --- | --- | --- | --- | --- | --- | --- | --- | --- | --- | --- | --- | --- | --- | --- | --- | --- | --- | --- | --- | --- | --- | --- | --- | --- | --- | --- | --- | --- | --- | --- | --- | --- |
|  |  | 8 | | | 10 | | | 16 | | | 31 | | | 69 | | | 71 | | | 113 | | | 115 | | | 120 | | | 140 | | | 149 | | | 164 | | | 194 | | | 269 | | | 299 | |  |
| CCV/Thailand/  2020 | MZ826142 | Q | | | V | | | G | | | P | | | K | | | **C** | | | V | | | **K** | | | N | | | A | | | F | | | T | | | **S** | | | **G** | | | Y | |  |
| CCV/Argentina/  2016 | MK033608 | Q | | | V | | | G | | | P | | | Q | | | T | | | P | | | R | | | N | | | S | | | Y | | | T | | | Q | | | P | | | H | |  |
| CCV/USA/ 2011 | KC241984 | Q | | | G | | | G | | | P | | | R | | | T | | | P | | | S | | | N | | | S | | | Y | | | T | | | D | | | P | | | H | |  |
| CCV/Germany/ 2014 | KT283604 | Q | | | G | | | G | | | P | | | Q | | | T | | | P | | | R | | | N | | | S | | | Y | | | T | | | E | | | S | | | H | |  |
| CCV/China/ 2016 | MF797786 | Q | | | G | | | G | | | P | | | K | | | A | | | V | | | R | | | N | | | A | | | F | | | T | | | N | | | S | | | H | |  |
| CCV/USA/ 2015 | MF457592 | Q | | | G | | | G | | | P | | | R | | | T | | | P | | | S | | | N | | | S | | | Y | | | T | | | E | | | P | | | H | |  |
| CCV/Italy/ 2013 | KT734823 | | | Q | | | G | | | G | | | P | | | R | | | T | | | P | | | S | | | N | | | S | | | Y | | | T | | | D | | | S | | Y | |
| CCV/KZ_2024 | | | **H** | | | **V** | | | **G** | | | **P** | | | **K** | | | **C** | | | **V** | | | **R** | | | **N** | | | **A** | | | **Y** | | | **T** | | | **S** | | | **G** | | | **H** | |
